# Financial Barrier against Access to Diagnostic Procedures among Enteric Fever Suspects in Highly-endemic Areas of China

**DOI:** 10.3329/jhpn.v28i1.4523

**Published:** 2010-02

**Authors:** Wen Xu, Virasakdi Chongsuvivatwong, Lin Lu, Xiao-Qing Fu

**Affiliations:** ^1^ Yunnan Provincial Centers for Disease Control and Prevention, 158 Dongsi Street, Kunming, Yunnan 650022, China; ^2^ Epidemiology Unit, Faculty of Medicine, Prince of Songkla University, HatYai 90110, Thailand

**Keywords:** Cross-sectional studies, Diagnosis, Laboratory, Haemoculture, Health expenditure, Health insurance, Healthcare, Typhoid, China

## Abstract

There is currently no public financial system that fully covers enteric fever suspects in China. This study aimed at documenting the level of access to definitive diagnostic procedures, especially haemoculture, for these patients and examining the effect of health insurance on access to such care. A hospital-based cross-sectional study was conducted in six counties of Yunnan province, using a structured questionnaire and data extraction from medical records. In total, 714 subjects were recruited. Chi-square test and logistic regression were employed for analysis of data. The majority of the subjects were young adults (52%) and farmers (55%) from low-income families (49%). Only 407 (57%) could afford haemoculture routinely advised by their doctors. Of these, 123 (30%) had haemoculture positive for *Salmonella* Typhi. After adjustment for income, not getting haemoculture was marginally associated with percentage of reimbursement from the insurance (p value for trend=0.047). Illiteracy was also an independent risk factor for this outcome. The poor coverage of haemoculture for patients suspected of having enteric fever in this endemic area was due to financial barrier. The current health-insurance system inadequately relieved the problem. Further financial reform to help patients suspected with enteric fever is required.

## INTRODUCTION

Nowadays, typhoid fever and paratyphoid (enteric fever in short) are rare in industrialized countries. However, they remain a serious health problem in the developing world. According to the best global estimates, at least 16 million new cases are diagnosed each year, with 600,000 deaths. Areas where the incidence reaches more than 100 cases per 100,000 people per year are considered to be endemic (1).

China has a relatively low incidence of enteric fever, ranging from two to five cases per 100,000 people per year. Yunnan, an underdeveloped province of southwestern China (gross domestic product [GDP] per capita of Yunnan was US$ 1,273 while the average GDP of China was US$ 2,280 in 2007) has the highest incidence of approximately 25 per 100,000 people per year in the nation (2). In 8–9 endemic counties of Yunnan, the rates rank the highest in China, reaching 100–375 per 100,000 people per year and contributing a total of 5,000 to 10,000 cases annually.

The absence of specific symptoms or signs makes the clinical diagnosis of this disease inaccurate. The definitive diagnosis of enteric fever depends on the isolation of *Salmonella* Typhi or *S.* Paratyphi from blood, bone-marrow, or a specific anatomical lesion (3). In practice, blood culture (haemoculture) is considered a minimum standard. Definitive diagnosis dictates adequate treatment of patients. Confirmed cases of enteric fever must be treated with efficacious antibiotic for at least 10 days to avoid relapse and carrier status. From a public-health point of view, accurate data are essential for priority setting, planning, and evaluation of various public-health measures. Proper diagnosis and treatment prevent carriers and promote public safety (4).

In developing countries, diagnoses of enteric fever are often made without cultures, especially in rural hospitals, due to lack of necessary facilities (5, 6). From the Disease Information System of Yunnan, 40–50% of reported cases did not have blood culture done, although 80% of enteric fever cases were reported from hospitals at the county level or above where the test is available. The underlying cause of this problem is expected to be the health-financing system. Provincial price bureau fixes the hospital charge for haemoculture at RMB 80 (or approximately US$ 11) but the patient must pay this fee.

In 2007, the Yunnan province had 129 counties with a total population of 44.8 million, 70% of whom were farmers. There are three main health-insurance schemes in China: the New Rural Cooperative Medical Scheme (NRCMS), the Urban Worker Medical Insurance (UWMI), and private insurance. In rural areas, the NRCMS is most popular. It is a voluntary scheme with an annual premium of RMB 10 collected from each farmer participating and a subsidy of RMB 20 obtained from the provincial and central governments. This scheme began in 2005, and the coverage was 85% in 2007. The scheme does not cover any diagnostic procedures for outpatients (7). Persons in the same family share the same family reimbursement account, which has a fixed limit for outpatient services. The scheme usually reimburses 10% of outpatient drug-fees at the village clinics and township hospitals, with a maximum limit equal to the family premium. Reimbursement at the outpatient department of the county hospital is not possible. The UWMI, which involves a government subsidy added to a small amount of premium deducted from staff salary, covered only 7.8% of Yunnan's population in 2007 (8). This scheme reimburses a high percentage of the costs of diagnostic procedures and drugs. Private medical insurance is rare and usually does not have reimbursement for outpatient services. In practice, any reimbursement from the insurance will be directly paid to the hospital and deducted from the patient's bill.

Under the local circumstance that enteric fever is endemic and laboratory tests are available but not fully used, it is important to know the proportion of suspected patients not getting the proper test and to examine the extent of financial constraints of the patient's family and the effect of insurance on access to diagnostic procedures.

The objectives of this study were to (a) document the proportion of enteric fever suspects who had not haemoculture done and (b) examine the effect of health insurance on access to this diagnostic procedure after adjustment for income and other confounders.

We expect that the results of the study will provide evidence to the policy-makers for taking decision on control measures and insurance planning to modify the scheme to improve such care.

## MATERIALS AND METHODS

### Study design and setting

This is a hospital-based cross-sectional study with face-to-face interview using a structured questionnaire and data extraction from medical records.

The study was conducted in six counties in Yunnan where the incidence of enteric fever is high. According to recent demographic data, there were approximately four million people residing in these areas (8). The incidence rates in these areas ranged from 100 to 375 per 100,000 people per year. Six study hospitals at the county level and 12 at the township level were selected based on the fact that their total contribution of reported enteric fever cases was around 80% of the whole province. All hospital administrations agreed to participate in the study. They also had facilities for examination of enteric fever, including haemoculture, the results of which are under a quality-control process checked by prefecture clinical laboratories.

### Definition of an enteric fever suspect

According to the World Health Organization and the Chinese national guidelines, an enteric fever suspect is a patient with fever (38 °C and above) which has lasted for at least three days, with symptoms of headache, malaise, disturbances of bowel function (constipation in adults, diarrhoea in children) and residing in endemic areas during an endemic season (3, 9).

### Study population and sample

The study population is all suspected cases of enteric fever in endemic areas of Yunnan province.

**Case ascertainment:** All patients presenting with fever were screened at the outpatient department for eligibility. To be eligible for the study, subjects must have been a native resident who had fever for at least three days before coming to the hospital without any specific symptom or sign of localized infection. Patients attending for their second or subsequent visit for the same episode of fever were excluded.

### Sample size

Based on the Disease Information System of Yunnan, around 50% of the enteric fever patients had no haemoculture confirmed. We assumed that half of our subjects would not be able to pay for the test. Among those who did pay, the prevalence of insurance would be 80% compared to 60% among those not paying. With a significance level of 0.05 and a power of 90%, the total sample size required was calculated to be 238. However, as the enteric fever-control programme required to have a broader picture of Yunnan province, the sample size was increased to 700 so that more than 100 cases were selected from each of the six study counties.

### Variables and collection of data

The main outcome variable is whether the patients received haemoculture. The main explanatory variable is percentage of medical expense reimbursable from the insurance system. Family-income is the key adjustment factor. Other variables included type of hospital and demographic characteristics, such as age, sex, education, and occupation. Data were collected from May 2007 to September 2008. Before data collection, a pilot study was conducted in a hospital. Twenty suspected cases of enteric fever were interviewed. The questionnaire was discussed with clinic doctors and CDC staff and then revised. Two full-time research assistants were employed for each study hospital. All 36 research assistants from the 18 study hospitals were trained to be familiar with the definition of suspected case of enteric fever, criteria of inclusion and exclusion, data-collection procedures, and interview methods. The outpatient services were arranged so that the attending physician took the clinical history and conducted physical examination of the patient. If the patient was eligible for the study, he/she would be given an explanation by the doctor about the necessity of the haemoculture before he/she decided to pay or not pay to have the test done. The research assistant then approached the patient for informed consent and interviewed them about all medical fees which may or may not include the haemoculture test. The haemoculture test was done on payment. The research assistant who followed the patient recorded the real payment of the patient from the bill and conducted a further interview for obtaining information, such as insurance type and reimbursement status, before the patient proceeded to the next section (i.e. laboratory or ward) of the hospital.

### Analysis of data

Background characteristics of the patients were described by frequency, percentage, median and interquartile range, for each type of hospital. Access to blood culture, total median expense incurred from the current illness and percentage of the medical expense reimbursable from the insurance were similarly tabulated. To assess the effect of income and insurance, the fraction and percentage of access to blood culture was computed for each combination of income and insurance reimbursement category. Finally, to adjust for other possible confounders, logistic regression predicting no access to blood culture was carried out with these two main factors and other significant covariates in the model. The final significance level was set at 0.05.

The ethics committees of the Yunnan Provincial Centers for Disease Control and Prevention (Yunnan CDC), and the Faculty of Medicine, Prince of Songkla University approved the study. Permission was also obtained from the hospital.

## RESULTS

During the study period, 714 subjects were recruited, of whom 234 were from the county hospitals and 480 from the township hospitals.

Table 1 presents the background characteristics of the subjects by type of hospital. The sample was preponderant by young adults, males, Han ethnicity, and farmers with a relatively low level of education, and 53.5% of the subjects were not covered by any type of insurance for the current medical service. The overall median value for annual per-capita income of the family was RMB 2,500, which was close to that of local farmers. Compared to patients from the county hospitals, those from the township hospitals were more likely to belong to a minority ethnic group, live in a rural area, have a larger family size, have a lower level of education, and lower annual per-capita income of family but covered by medical insurance.

**Table 1.** Background characteristics of subjects (n=714)

Characteristics	Frequency (%) and median (IQR)		
	County hospital (n=234)	Township hospital (n=480)	Total (n=714)
Age (years) (median*, IQR)	29.7 (19.7, 39)	27.2 (12.6, 40.2)	28.3 (15.6, 40)
Gender			
Male	122 (52.1)	266 (55.4)	388 (54.3)
Female	112 (47.9)	214 (44.6)	326 (45.7)
Ethnicity			
Han	184 (78.6)	217 (45.2)	401 (56.2)
Dai/Hani/Yi/Hui/others	50 (21.4)	263 (54.8)	313 (43.8)
Occupation, frequency (%)			
Farmers	115 (49.1)	280 (58.3)	395 (55.3)
Workers/staff/businessmen	48 (20.5)	31 (6.5)	79 (11.1)
Students	47 (20.1)	97 (20.2)	144 (20.2)
Preschool children	11 (4.7)	69 (14.4)	80 (11.2)
Others (housewife/jobless)	13 (5.6)	3 (0.6)	16 (2.2)
Patient's family residence			
Urban	70 (29.9)	29 (6)	99 (13.9)
Rural	164 (70.1)	451 (94)	615 (86.1)
Family-size (median, IQR)	4 (3, 4)	4 (4, 5)	4 (4, 5)
Education achieved (%)			
None	19 (8.1)	54 (11.2)	73 (10.2)
Preschool	11 (4.7)	69 (14.4)	80 (11.2)
Primary school	68 (29.1)	177 (36.9)	245 (34.3)
Middle school	74 (31.6)	139 (29)	213 (29.8)
High school/vocational	45 (19.2)	36 (7.5)	81 (11.3)
University	17 (7.3)	5 (1)	22 (3.1)
Annual per-capita income (RMB) of family† (median, IQR)	2,250 (1,400, 3,750)	2,106.7 (1,000, 5,000)	2,225 (1,250, 4,733.3)
133–1,250	36 (15.4)	165 (34.4)	201 (28.2)
1,251–2,500	51 (21.8)	77 (16)	128 (17.9)
2,501–6,000	65 (27.8)	94 (19.6)	159 (22.3)
6,001–45,000	82 (35)	144 (30)	226 (31.7)
Covered by any type of medical insurance, frequency (%)	43 (18.4)	289 (60.2)	332 (46.5)

*IQR=Inter-quartile range;

†Quartile of whole dataset

Table 2 presents access to blood culture and the financial situation of subjects at the current hospital. Fifty-seven percent of the suspects received haemoculture. The median value of medical expenses was RMB 264, which was higher than 10% of the median of annual per-capita income of the study families (RMB 2,500). While the median medical expense for the current illness was similar in the subjects from the two types of hospitals, the percentage of reimbursement was much lower at the county hospital where the percentage of receiving blood culture was contradictorily higher.

**Table 2.** Access to blood culture and financial situation of subjects at the current hospital*

Characteristics	Frequency (%) and median (IQR)	
	County hospital (n=234)	Township hospital (n=480)	Total (n=714)
Access to blood culture	150 (64.1)	257 (53.5)	407 (57.0)
Medical expense for illness† (median, IQR)	263.9 (190.8, 389)	264 (176.7, 460.5)	264 (180.1, 424.2)
Percentage of reimbursement for all types of payment (%)			
0–10	193 (82.5)	196 (40.8)	389 (54.5)
11–50	21 (9)	240 (50)	261 (36.6)
51–100	20 (8.5)	44 (9.2)	64 (9)

*All financial values are in RMB;

†Current medical expense for current illness in RMB (median);

IQR=Inter-quartile range

Altogether 407 patients had their haemoculture done. Of them, 123 (30%) had positive results for *S.* Typhi. Of the haemoculture-positive patients, 98 were hospitalized, 24 were treated as outpatients, and one patient ran out of money, and he bought medicines from a drug-store and stayed at home. Of 284 patients who had a negative haemoculture results, 28 were hospitalized, and 256 were treated at the outpatient service.

Table 3 presents the number of patients not getting haemoculture/total (percentage) by per-capita family-income quartile and percentage of medical expense reimbursable from the insurance. Patients from a low-income family and those having a low percentage of reimbursement were less likely to get haemoculture. Of these two factors, family-income had a stronger effect. Of the 168 subjects in the top income quartile, all had haemoculture done. In contrast, as much as a quarter of the 64 subjects who could get more than half of the medical expenses reimbursed did not receive such care.

**Table 3.** Number of patients not getting haemoculture/total (%) by per-capita family-income quartile and percentage of medical expense reimbursable from the insurance

Percentage of reimbursement	Per-capita family-income quartile				Total
	1st	2nd	3rd	4th	
0–10	118/128 (92.2)	64/98 (65.3)	11/104 (10.6)	0/59 (0)	193/389 (49.6)
11–50	60/65 (92.3)	38/57 (66.7)	0/60 (0)	0/79 (0)	98/261 (37.5)
51–100	5/8 (62.5)	11/17 (64.7)	0/9 (0)	0/30 (0)	16/64 (25.0)
Total	183/201 (91.0)	113/172 (65.7)	11/173 (6.4)	0/168 (0)	307/714 (43.0)

Table 4 presents the univariate analysis of predictors for access to haemoculture. Of nine independent variables showing significant association with the outcome in the univariate analyses, only three were included in the final logistic regression model. Factors which were not significant in logistic regression were age, sex, family size, ethnicity, occupation, and family residence. Family-income had the strongest independent effect. Using the lowest quartile as the reference group, the second quartile had less than one-fifth the odds of having the problem of not receiving haemoculture. The third quartile further had only six in a thousand times the risk among the poorest. Since none of the richest group had the problem of not receiving haemoculture, the odds ratio was too low to be computed. Education level was also a significant independent predictor. Illiterate subjects had the highest odds of not getting haemoculture. There was no dose-response relationship among the remaining subjects with different levels of education. The percentage of reimbursement was not significant (p=0.13) but was marginally so when a test for linear trend was applied (p=0.047). This suggests that insurance has a marginally important role in helping the patient to receive haemoculture.

**Table 4.** Predicting factors for not getting haemoculture among enteric fever suspects

Variable	Univariate analysis			Logistic regression	
	Getting haemoculture (n=407)	Not getting haemoculture (n=307)	Chi-square* p value	Adjusted OR (95% CI)	p (LR-test)
Annual per-capita income (RMB) of family			<0.001		<0.001
132–1,250	18 (4.4)	183 (59.6)		1	
1251–2,500	59 (14.5)	113 (36.8)		0.191 (0.106–0.344)	
2,501–6,000	162 (39.8)	11 (3.6)		0.006 (0.003–0.014)	
6,001–45,000	168 (41.3)	0 (0)		−	
Percentage of reimbursement for all types of payment			<0.001		0.1324¶
0–10	196 (48.2)	193 (62.9)		1	
11–50	163 (40)	98 (31.9)		0.718 (0.42–1.228)	
51–100	48 (11.8)	16 (5.2)		0.416 (0.163–1.06)	
Education level achieved (%)			<0.001		0.0019
Illiterate	23 (5.7)	50 (16.3)		1	
Primary school	132 (32.4)	113 (36.8)		0.169 (0.057–0.497)	
Middle	131 (32.2)	82 (26.7)		0.153 (0.051–0.453)	
High school+	121 (29.7)	62 (20.2)		0.197 (0.064–0.611)	
Age (years)			0.03	NS§	
0–19	133 (32.7)	101 (32.9)			
20–39	175 (43)	126 (41)			
40–59	84 (20.6)	53 (17.3)			
60–85	15 (3.7)	27 (8.8)			
Sex			0.103	NS	
Male	220 (43.7)	284 (56.3)			
Female	233 (49.1)	242 (50.9)			
Family size			<0.001	NS	
1–4	289 (71)	172 (56)			
5–7	118 (29)	135 (44)			
Ethnicity			0.046	NS	
Han	215 (52.8)	186 (60.6)			
Dai/Hani/Yi/Hui/others	192 (47.2)	121 (39.4)			
Occupation			<0.001	NS	
Farmers	203 (49.9)	192 (62.5)			
Urban employees	71 (17.4)	8 (2.6)			
Unemployed	133 (32.7)	107 (34.9)			
Family residence			<0.001	NS	
Urban	80 (19.7)	19 (6.2)			
Rural	327 (80.3)	288 (93.8)			

*Chi-square test;

¶Value for linear trend=0.047;

§Not statistically significant and not included in the model;

CI=Confidence interval;

LR=Likelihood ratio;

NS=Not significant;

OR=Odds ratio

## DISCUSSION

In these endemic areas for enteric fever, the potential cases of enteric fever were young adult farmers from low-income families. Unfortunately, only about a half could have haemoculture routinely advised by the doctors. The problem of not having this test was more serious at the township hospital despite the higher percentage of insurance coverage. Of the subjects tested, 30% had the organism detected. Despite this seriousness, a large proportion of subjects was treated without admission. After adjustment for family-income, the effect of insurance reimbursement was only marginal. Of all other demographic variables, literacy of the patient also had an independent effect on not having the test done.

Our findings that suspects of enteric fever were farmers from poor families are similar to those in a previous report from India (10), Indonesia (11), and Bangladesh (12). Enteric fever is transmitted through contamination of food and water and is associated with poverty and poor hygiene. The poor who are at a higher risk than the rich also received less complete diagnostic care when they were suspected to have enteric fever from the communicable disease.

Medical care and financial burden to the patients at the township and county hospitals are quite different. The popular health insurance—NRCMS—partly reimbursed medical fees only when the service was at the township hospital. Patients at a county hospital were, therefore, less likely to be assisted by the insurance. However, the percentage of patients having a blood culture done was higher in the county hospital. This was due to the higher income of patients served by this level of hospital. The difference in income confounded the effect of the hospital. After adjustment for income, the effect of hospital was not significant. The implication of this finding was that this diagnostic care is differently accessed by the patients mainly due to the limitation of income of the patient, not due to the hospital setting.

Lack of haemoculture test among 43% of the subjects is a finding of the serious consequences. Obtaining a definitive diagnosis of enteric fever is more cost-effective than providing empirical treatment because it can cut down unnecessary treatment given to non-cases and emphasize proper antibiotic use, which needs to be as long as 10 days (9, 13). However, 30% of those not having the test might also have *Salmonella* in their blood. With inadequate treatment, a patient may end up with antibiotic resistance, become a chronic carrier, develop severe complications, and consequently die (14).

The very steep slope of dose-response relationship between the family-income and the chance of having blood culture elaborates the financial barrier from the out-of-pocket fee for service system. Economists have developed a technique to evaluate worth for money of various health services based on interviews with a sliding scale of willingness to pay (15–24). Ability-to-pay—another economic term comparing the medical expense incurred with annual family expenditure—has also been used for accessing financial burden (25). Our study did not include those variables since we considered that blood culture is a life-saving process for serious infection and should not have a monetary value attached to the poor patient. The cost (RMB 80) of this service is not going to cripple the family. Access to this care is, therefore, more a humanitarian than financial concern. However, a suitable financial management system must exist to enable the service. Health insurance is almost universally adopted as a financial safety-net for most illnesses. Unfortunately, health insurance in the study area had a poor coverage and inadequate reimbursement, which has only a marginal effect on access to blood culture. The insurance system, therefore, does not guarantee full access to this necessary care. Moreover, our results indicate that the illiterate tended not to get the service. Leaving the decision to the poor who are sick and inadequately educated is, thus, not justifiable.

Inability to have definitive diagnostic tests of communicable disease is not uncommon in developing countries. A study in India showed that blood culture for enteric fever was also expensive (26). In Cambodia, citizens are reported to be too poor to afford the fee of blood test for a diagnosis of dengue (27).

Inability to receive hospital services in China has recently become an important public-health and social problem. Hospital costs rose from 20% of total health expenditure in 1980 to 59% in 2000 and slightly dropped to 49% in 2006 (28). More than 35% of urban and 43% of rural households have difficulty in affording healthcare costs (29).

Our study was conducted at the county- and township-level hospitals where rural medical insurance is involved. It provides a good illustration of the financial pitfalls on communicable disease control. However, since the study areas are endemic for enteric fever, the conclusion may not be generalized to areas where epidemiological settings are different.

In conclusion, patients affected by febrile illness in this study face a financial barrier to obtain proper diagnosis for endemic enteric fever. The existing health insurance is inadequate. Better financial management is needed to improve the situation.

## ACKNOWLEDGEMENTS

This research is a part of the first author's thesis to fulfill the requirement for PhD in epidemiology at Prince of Songkla University., Thailand. The authors thank Qujing, Xishuangbanna, Honghe, Yuxi prefecture-level Centers for Disease Control and Prevention (CDC) and Luoping, Shizong, Menghai, Mengla, Jianshui, Mile, Mengzi county-level CDC doctors for carrying out field work. Financial support was provided by Yunnan Provincial CDC.
